# Site-specific electrical contacts with the two-dimensional materials

**DOI:** 10.1038/s41467-020-17784-3

**Published:** 2020-08-07

**Authors:** Lok-Wing Wong, Lingli Huang, Fangyuan Zheng, Quoc Huy Thi, Jiong Zhao, Qingming Deng, Thuc Hue Ly

**Affiliations:** 1 Department of Applied Physics, The Hong Kong Polytechnic University, Kowloon, Hong Kong China; 2 Polytechnic University of Hong Kong Shenzhen Research Institute, Shenzhen, China; 3 Department of Chemistry and Center of Super-Diamond & Advanced Films (COSDAF), City University of Hong Kong, Kowloon, Hong Kong China; 4 City University of Hong Kong Shenzhen Research Institute, Shenzhen, China; 5 Physics department and Jiangsu Key Laboratory for Chemistry of Low-Dimensional Materials, Huaiyin Normal University, 223300 Huaian, China

**Keywords:** Materials science, Electronic properties and materials, Two-dimensional materials

## Abstract

Electrical contact is an essential issue for all devices. Although the contacts of the emergent two-dimensional materials have been extensively investigated, it is still challenging to produce excellent contacts. The face and edge type contacts have been applied previously, however a comparative study on the site-specific contact performances is lacking. Here we report an in situ transmission electron microscopy study on the contact properties with a series of 2D materials. By manipulating the contact configurations in real time, it is confirmed that, for 2D semiconductors the vdW type face contacts exhibit superior conductivity compared with the non-vdW type contacts. The direct quantum tunneling across the vdW bonded interfaces are virtually more favorable than the Fowler–Nordheim tunneling across chemically bonded interfaces for contacts. Meanwhile, remarkable area, thickness, geometry, and defect site dependences are revealed. Our work sheds light on the significance of contact engineering for 2D materials in future applications.

## Abstract (web summary)

Here, the authors use in situ transmission electron microscopy to measure the interface properties of electrical contacts with MoS_2_, ReS_2_, and graphene, and find that direct quantum tunnelling across van-der-Waals-bonded interfaces is more favourable than Fowler–Nordheim tunnelling across chemically bonded interfaces.

## Introduction

Since graphene was exfoliated by Geim et al.^1^, two-dimensional (2D) materials, such as transition metal dichalcogenides synthesized by either top-down^2,3^ or bottom-up^4–6^ methods, have drawn enormous attentions because of their attractive properties^7–12^ and potential applications^13–16^. Among all the applications, transistors are most noteworthy because of their key roles in electronic devices currently. The Moore’s Law^17^—the size of transistors would be half and the number of transistors in an integrated circuit would be doubled every year, nowadays is slowing down to around every two years or longer. Nevertheless, 1-nm gate length^18^ and 1-nm channel width^19^ have been reported. With the trend of miniaturization, to deal with these nanoscale or even sub-nanoscale transistors and devices in near future, electrical contact, especially the metal-semiconductor (M-S) contact becomes an essential topic.

In conventional bulk M-S contacts, the interfacial hybridization or bonding are usually produced and considered beneficial for contact performances^20,21^. For example, the fluorinated graphene assisted top contact^22^ and graphene edge contact^23^ exhibited low contact resistance and high mobility. Very recently, an entire vdW-type (non-chemical) contact for the 2D transition metal dichalcogenide with ultra-clean and atomically sharp interfaces was reported, showing excellent conductivity^20^. It is thus crucial to understand the underlying transport mechanism to produce high quality contacts for the 2D materials.

Other than the highlighted performances of 2D devices^13–16^, contact geometric effects that improve the performance of devices are also reported^20–26^. For graphene, the edge-type contacts were found more favorable than the face contacts (or top contacts)^25–27^. Besides, the previous studies mostly investigated the electrical transport using mesoscopic symmetric device structures^15,20,21,24–26^ and in face contact configurations^13,14,16,28–30^, but the specific atomic-scale structures at the contact and electrical measurements remain uncorrelated. Here, we employ in situ transmission electron microscopy (TEM), and directly correlated the transport characteristics with the nanoscale contact configurations. Surprisingly, the electron direct tunneling (DT)^31^ across the nanoscale vdW contacts exhibit higher conductivities than the Fowler–Nordheim (FN)^32^ tunneling across chemically interacted contacts. 2D MoS_2_, ReS_2_, and graphene are probed by different contact areas in face or edge contacts. The thicknesses of 2D flakes, contact area, contact configurations, and atomic defects are found crucial for the 2D contact performances. Except from the structural information provided, the other benefits from our in situ TEM works also include: (1) We can manipulate the contact configuration on the same 2D material samples, and directly compare contacts at different sites; (2) Strain can be applied on the 2D materials when contacting; (3) The point-like contact configuration here in our experiment avoid the shortcoming of previous reports^25,26,33,34^, which have to consider more than one contacts in device measurements and in the *I*–*V* result explanations.

## Results

### In situ TEM setup for electrical measurements

**Fig. 1:**
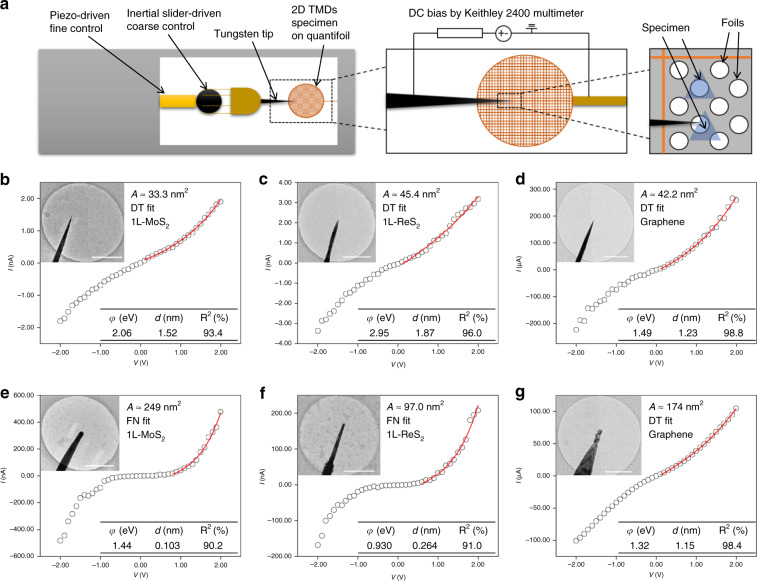
Current–voltage measurements of the W-CVD grown 2D material contacts. **a** Schematic of experimental setup. **b**–**d***I*–*V* measurements of CVD grown MoS_2_, ReS_2_, and graphene monolayer with small (*A* < 50 nm^2^) W probes, respectively. The inset show TEM images of the contact morphology and scale bars = 0.5 µm. The measured contact area (*A*), fitting model and the fitting parameters are also shown. The solid lines indicate the fitted range. **e**–**g***I*–*V* measurements of CVD grown MoS_2_, ReS_2_, and graphene monolayer with large (*A* > 95 nm^2^) W probes, with inset TEM images of the contact morphology, scale bars = 0.5 µm. The complete fitting curves can be seen in Supplementary Fig. 2.

Here we report the electrical transport across various types of contacts of 2D materials (MoS_2_, ReS_2_, and graphene). The sample preparations are presented in Methods section. In situ TEM-scanning tunneling microscopy (STM) technique we applied is a direct method to study the structure-property relationship and empower the atomic-scale understanding of the electrical performances of materials^35–37^. Figure 1a shows the experimental scheme of our room temperature in situ TEM-STM setup (also see Supplementary Fig. 1), with programmable bias voltage and current measurement module (see “Methods” section). A homemade piezo-controlled tungsten (W) STM tip provided the sub-nanometer 3-axis control, which manipulated the contacts with the free-standing chemical vapor deposition (CVD) grown 2D membranes or mechanically exfoliated 2D materials. Tungsten is chosen for contact material due to its low thermal expansion, high melting point/modulus and non-corrosive properties, hence often used for metallic interconnects and contact windows in nanoscale devices, such as in the fin field-effect transistor (FinTFT)^33^. The point-like contacts between the STM tip and the 2D materials throughout our experiments ensured that the *I*–*V* data can directly reflect the transport behavior of the focused contacts.

### Measurement on free-standing CVD grown 2D membranes

Free-standing CVD grown single layer (1L) MoS_2_, ReS_2_, and graphene were probed and measured first. The STM tips was carefully moved towards the sample until solid contacts were made, and each set of *I*–*V* data for the contacts was multiple-checked to be stable and reproducible. The *I*–*V* measurement results and the face contact morphologies can be seen in Fig. 1b–g. In addition, fitted curve, fitting parameters and measured contact area are also shown (Fig. 1b–g). Two models, viz. direct tunneling (DT)^38^ and Fowler–Nordheim (FN) tunneling^32^, were used to fit the experimental data. In the perspective of MoS_2_ and ReS_2_ contacts (Fig. 1b, c, e, f), an approximately linear *I*–*V* curve can be seen in smaller area contacts (<50 nm^2^) and can only be fitted by the DT model (Fig. 1b, c), while rectifying and nonlinear behaviors can be observed in larger area contacts (>95 nm^2^) and can be exclusively fitted by the FN tunneling (Fig. 1e, f). The contact area measurements can be seen in see “Methods” section.

These exclusive fitting results imply that the barrier shape is closer to trapezoidal type for smaller contacts and triangular type for larger contacts, respectively. The characteristic linear fit of DT is ${\mathrm{ln}}(I/V^2) \propto {\mathrm{ln}}(1/V)$ and that of FN tunneling is ${\mathrm{ln}}(I/V^2) \propto (1/V)$, which are demonstrated in Supplementary Fig. 2, respectively. Comparing the fitting parameters with two transport mechanisms, the barrier height (*φ*) and barrier width (*d*) are larger in the DT cases (small contact area). In terms of 2D semiconductors (MoS_2_, ReS_2_), for DT cases, the barrier heights are within 2.06 to 2.95 eV and barrier thicknesses are within 1.52–1.87 nm; For FN cases, the barrier heights are within 0.930–1.44 eV and barrier thicknesses are within 0.103–0.264 nm. The barrier widths are both narrow enough for tunneling (<5 nm)^39^.

In terms of semi-metal graphene, different from the MoS_2_ and ReS_2_ contacts, DT behavior with much higher conductivity was observed even in large area contacts. The *I*–*V* data exhibit strongly linear associations, which can be well fitted with the DT model, even in different contact areas (Supplementary Fig. 2). The barrier heights are 1.49 and 1.32 eV, and barrier widths are 1.23 and 1.15 nm as shown in Fig. 1d, g, respectively. The fitting characteristics can be seen in Supplementary Fig. 2. The fitting parameters are similar to the previous MoS_2_ and ReS_2_ cases, whose potential barriers are larger than 1 eV and barrier widths are between 1 and 2 nm.

The W probe could further stress onto the membranes to check whether the strain affects the transport mechanism and the band structure. The strain dependent results were all analyzed on the face contacts, by compressing the tip on the surface of suspended CVD grown samples. The contact area dependence effect has been excluded, thus the results solely correspond to the in-plane strain (Supplementary Fig. 3). The higher in-plane strain can reduce contact resistivity for graphene and MoS_2_ but increase the contact resistivity for ReS_2_. This effect might be attributed to the suppression of vdW contact barriers (for DT tunneling) in graphene and MoS_2_ and facile introduction of defects in ReS_2_ (such as lattice reconstruction) by strain.

### Measurement on mechanically exfoliated 2D samples

**Fig. 2:**
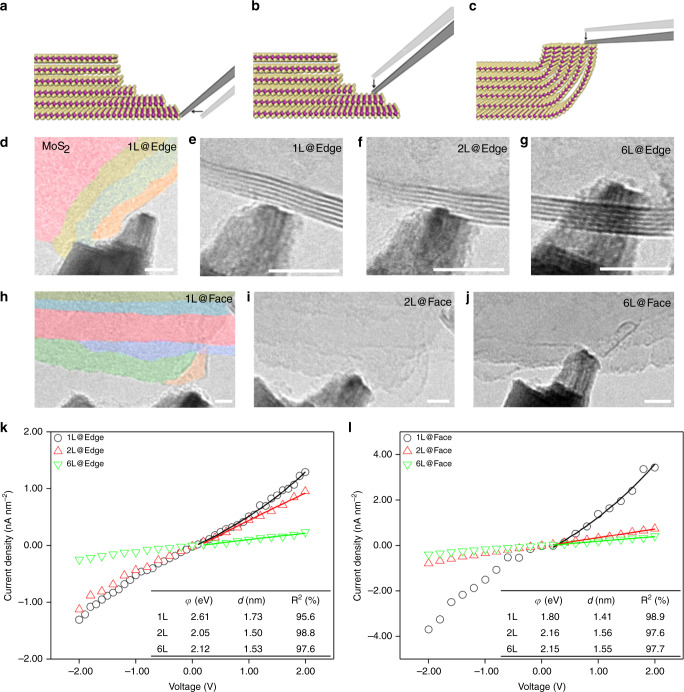
Electrical contacts of W-exfoliated MoS_2_. Side view illustrations of **a** 1L MoS_2_ edge contact, **b** 2L MoS_2_ face contact, and **c** 2L MoS_2_ edge contact. The yellow, purple, and white balls on **a**–**c** represent sulfur atoms, molybdenum atoms, and tungsten atoms, respectively. The black arrows in **a**–**c** indicating the moving direction of the W tip. TEM images of **d**, **e** 1L, **f** 2L, and **g** 6L edge contact. TEM images of **h** 1L, **i** 2L, and **j** 6L face contact. The color on **d**, **h** aims to enhance the contrast of the stair-like structure. Scale bars in **d**–**j** =5 nm. **k**, **l** Current density–voltage (*J*–*V*) plots of **e**–**g** and **h**–**j**, respectively. The solid lines indicate the fitted range. The inset table shows the fitting parameters and the fitting curves can be seen in Supplementary Fig. 3.

Besides the tests on the CVD grown specimens, mechanically exfoliated 2D MoS_2_ was studied as well. Some exfoliated MoS_2_ flakes have stair-like structure, which allow us to study the layer-dependent properties and contact geometric effect for the same flake. As two major contact configurations for the 2D materials, edge contacts and face contacts were unambiguously produced and identified as Fig. 2a–c (side view). The plane surfaces of the 2D flakes can be perpendicular (planar view) or parallel (edge-on view, edges were bended) to the viewing direction of TEM (Fig. 2c). The black arrows indicate the moving direction of the W tip. The W tip was gently moved and connected with the edges or surfaces for the two types of contacts. For contacts in planar view, the thickness (layer number) of contact position was confirmed by mechanically bending the edges into edge-on configuration, thus the layer numbers can be directly counted. All W-exfoliated MoS_2_ contacts were less than 50 nm^2^.

Corresponding TEM images captured from continuous in situ TEM manipulating process are shown in Fig. 2d–j. The false colors enhanced the thickness contrast in Fig. 2d, h, presenting the stair-like structure of the 6-vdW-layered (6L) MoS_2_ which enabled the W tip to contact with different number of vdW layer(s) by changing the contact positions, including the ultimate 1L contact. Figure 2d, e shows typical 1L edge contact and Fig. 2f, g display the bilayer (2L) and 6L edge contact, respectively. On the other hand, 1L, 2L, and 6L face contact are shown in Fig. 2h–j, respectively. The W tip was sufficiently sharp which provided atomic-accurate contact. The 2D (*x*–*y* plane) projection effect of TEM imaging may cause overlap between W tip and 2D flakes, however all the edge and face contacts in our experiments have been precisely controlled in 3D by the movable W tip so that the relative position (including *z*-direction) of the W tip and flake edge can be unambiguously determined (Supplementary Fig. 4, also see Supplementary Movie 1 for details). The contact formation can be confirmed by direct TEM observations or electrical signal (see “Methods” section). Simultaneously, *I*–*V* measurement for different contact configurations was carried out. The recorded *I*–*V* responses showed large variations for different contacts even at the same contact position of the same MoS_2_ flake, exhibiting the point-like contact governed transport behavior. The average potential difference of edge contacts and face contacts are 0.17 and 0.19 eV respectively, that also implies the edge contacts are slightly more stable than face contacts, that is facilitated by the chemical bonding (Supplementary Table 1).

The vdW and non-vdW-type contacts were primarily distinguished by the electrical measurements in ln(*I*/*V*^2^) ~ 1/*V*^40^. The DT behavior corresponds to the flat barriers, so called vdW gap on the interfaces. FN tunneling behavior corresponds to the chemically modified energy band realignments so they are determined as non-vdW type. Similar FN tunneling phenomena has been found in the non-vdW-type contacts^40^. The area dependence on the contact types clearly manifest the large area contacts (irrespective face or edge) are prone to have FN tunneling behavior, due to the presence of higher density of atomic defects (vacancies, edge irregularities) for larger contact areas. Albeit the TEM images sometimes cannot directly tell the vdW or non-vdW-type contacts apart, we did observe some good vdW contacts with two planer surfaces at the interfaces, which also coincide with its excellent DT behavior, such as shown in Supplementary Fig. 2.

In previous works, the electrical conductivities were usually higher for the contacts with larger number of layers^38,41^, irrespective of edge contact or face contact. However, our results demonstrated contrary that the electrical conductivities were higher for smaller number of layers, also irrespective of edge contact or face contact. It can be seen in the current density versus voltage (*J*–*V*) graph (Fig. 2k, l). Meanwhile, the current density is significantly higher in 1L face contact than in 1L edge contact against with the previous reports^25^. Besides, the current density in 1L face contact is approximately double compared with 1L edge contact that suggested the conductivity is lower when the number of chemical bonding (or defects) is increased.

The chemical contact only makes bonding with the defects locally. Based on our DFT calculations (Supplementary Fig. 5), if there are S vacancies in the basal plane of MoS_2_, the adsorption energy between W and MoS_2_ flake will be increased by 0.56 eV per atom, which is still relatively low, and it means the contacts can be easily detached at room temperature (basically, 0.7–0.8 eV interactions can be spontaneously overcome by thermal energy at 300 K in the time scale of 1 s). So, most of the contacts in our experiments are not hard to detach, irrespective of vdW type or non-vdW type. The chemical interactions between the W and defects of MoS_2_ can modify the electronic structure (Supplementary Fig. 5), more defective states are in presence.

In addition, in our experiments, a larger area chemical contacts can be made by applying high voltage bias (such as 10 V), which induced the significant joule heating effect at the contact, in this case, the interfaces (mainly the W tip side) were locally heated up to (or close to) the melting temperature and stronger (larger area) chemical bonding will be made, and more difficult to break (Supplementary Fig. 6). The chemical interaction in this case was much larger than the vacancy interactions above. The transport curve of this type of welded contacts exhibited significant rectifying behavior, and both directions have high energy barriers, thus, dominated by the thermionic field emission (Supplementary Fig. 6).

## Discussion

Overall, we found the chemical bonding will not be beneficial to the contact conductivity, due to the presence of pronounced electron scattering by the defects (Supplementary Fig. 7 and Supplementary Note 1). The Fermi level pinning at the defective states deep in the bandgaps can easily trigger FN barriers at the contacts (for 2D-TMDCs which are usually n doped). The flat, smooth and parallel vdW contact (normally the face contact) is better choice for making contacts with 2D materials, especially for the 2D-TMDCs. Accordingly, by the characteristic fit of direct tunneling (DT)^31^, the linear fit of ${\mathrm{ln}}(I/V^2) \propto {\mathrm{ln}}(1/V)$ was well agreed with the data of contact with exfoliated samples in 0.10–5.00 V (Supplementary Fig. 8). The fitted barrier widths (*d*) are between 1 and 2 nm, which coincide with the results of small contacts with CVD samples. The fitted barrier heights are from 1.80 to 2.61 eV. 42 results with different contact configurations can be seen in Supplementary Table 1.

**Fig. 3:**
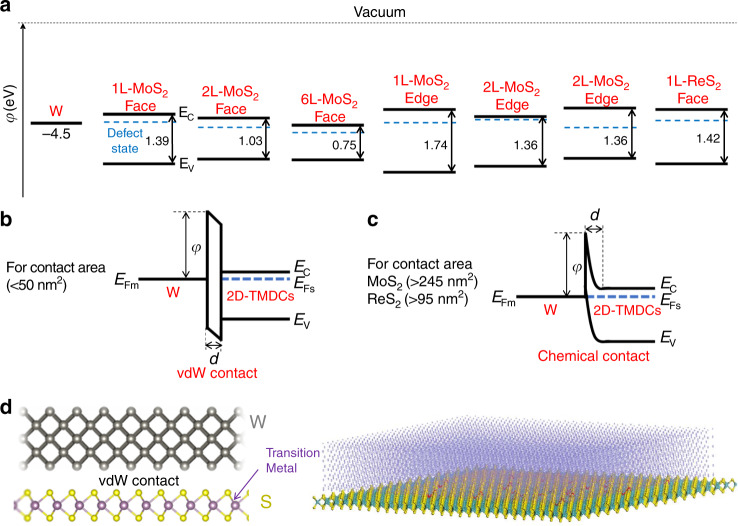
Band formation of the W-to-TMDCs electrical contacts. **a** Band alignment before W-2D-TMDCs contact obtained by DFT calculation. **b**, **c** Band formation of 2D-TMDCs after W nanotip contacts with different area. Detailed information in Supplementary Table 1 and Supplementary Fig. 2. **d** Illustration of the contact area effect (left: small contact; right: large contact) to the W-2D-TMDCs.

Summarizing the contacts with 2D semiconductors such as MoS_2_, ReS_2_, the density functional theory (DFT) was applied and the calculated band structures are illustrated (Fig. 3a–c, also see Supplementary Fig. 9 and Supplementary Note 2). The band formation of ReS_2_ is constructed by the references^42,43^. The measured DT/FN barrier heights are higher than the prediction of Schottky-type barrier due to interfacial band alignment^42,44–46^. Due to the abundant intrinsic sulfur vacancies of the 1L MoS_2_, the Fermi level is close to the conduction band minimum^47,48^, hence the lowest potential difference between forward bias and reverse bias is close 0 eV, due to the highly symmetric *I*–*V* behaviors (Fig. 1). A comprehensive understanding of the DT and FN tunneling behavior for the contacts should origin from the barrier shapes. VdW-type interfaces (non-chemical interfaces) normally possess flat barriers, while the chemical bonded interfaces possess triangle barriers due to the electronic hybridizations. For the face or edge contacts containing defect levels (Fig. 3a), the Fermi level pinning may alter the alignment of bands at the 2D material side. The defect states such as vacancies present in the 2D planes or on the 2D edges can pin the Fermi levels through the local charges, further elevate (at the peak) and attenuate (at the tail) for the contact barriers, which leads to FN tunneling. All our results suggest both small and large area contacts have the formation of the narrow potential barriers, for both forward and reverse directions.

Ultra-thin barrier is desirable for the quantum tunneling. The illustrations of band formation of the contacts and the related transport mechanism with respect to contact area can be seen in Fig. 3b, c. Based on our results, the critical contact area for DT-to-FN transition is 249 and 97.0 nm^2^ for MoS_2_ and ReS_2_, respectively. The degree of bonding formation is the critical difference between DT and FN tunneling. As confirmed by the high resolution TEM characterizations, the defects (like S vacancies) randomly distribute in 2D materials^49^. Therefore, large contact area has a higher probability to form chemical interactions, thus FN tunneling is rendered (Fig. 3d). In terms of the contact with CVD grown graphene, due to the intrinsic semi-metal property and the ultra-low atomic defect density, the chemical bonding probability is low, hence yielding vdW contacts and direct tunneling behavior.

**Fig. 4:**
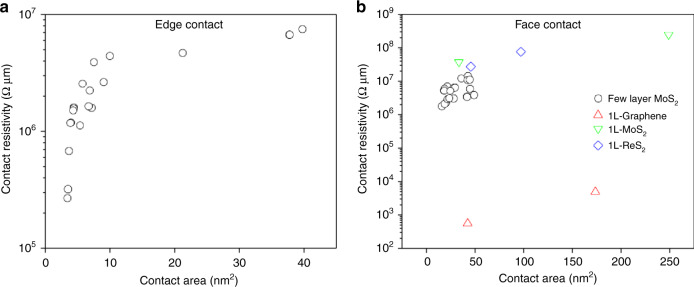
Measured contact resistivity by in situ TEM. **a** Edge contact and **b** face contact.

The contact resistivity (*ρ*) shows strong area dependency in different types of materials (Fig. 4a, b). Notably, contact resistivity of the transition metal dichalcogenides (TMDCs) contacts is from 2.67 × 10^5^ to 2.47 × 10^8^ Ω μm, which agree with the measurements of the device-based results in a reasonable range^20,21,34^. In agreement with the above argument on the defect-induced FN behavior, the exfoliated 2D materials possess less defects than CVD grown samples, hence the exfoliated samples predominantly have vdW-type contacts with DT behavior. Table 1 briefly summaries all the contact information exhibiting the evolution of the transport mechanism in these 2D contacts. The vdW-type contacts show much lower resistivity via DT tunneling than the non-vdW-type contacts via FN tunneling, which can be attributed to the homogeneous conducting channels, which are available in vdW contacts in comparison to the segregated conducting channels in chemical contacts and defect zones (non-conducting channels) with higher resistivity (Supplementary Fig. 7).

**Table 1:** Brief summary of all contacts.

Material	Measured contact area (nm^2^)	Model	Contact resistivity (Ω µm)
CVD MoS_2_	33.3	DT	3.81 × 10^7^
CVD MoS_2_	249	FN	2.47 × 10^8^
CVD ReS_2_	45.4	DT	2.78 × 10^7^
CVD ReS_2_	97.0	FN	7.68 × 10^7^
CVD graphene	42.2	DT	5.54 × 10^2^
CVD graphene	174	DT	4.90 × 10^3^
Exfoliated MoS_2_	<50.0	DT	2.67 × 10^5^–1.43 × 10^7^

**Fig. 5:**
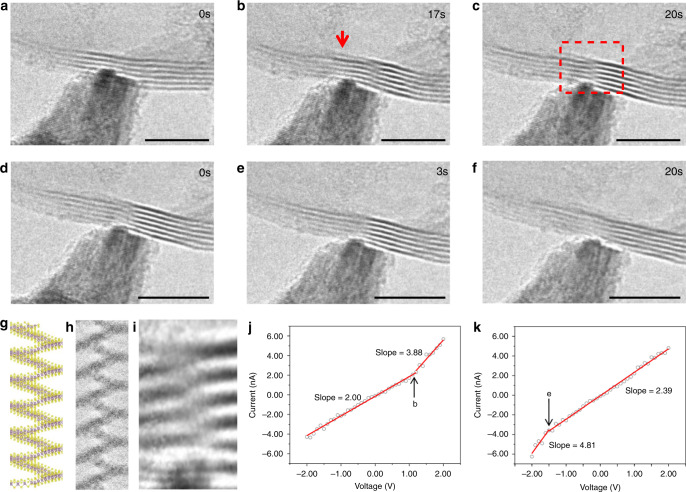
In situ observation of the formation/elimination of a single screw dislocation in the contact area. TEM images of screw dislocation appearing during measurement at **a** 0 s, **b** 17 s, and **c** 20 s. TEM images of screw dislocation disappearing during measurement at **d** 0 s, **e** 3 s, and **f** 20 s. **g** Atomic model, **h** simulated HREM image (by JEMS using multislice method), and **i** experimental image enlarged from the red dash box on **c** along zone axis [010]. **j** Current–voltage curve for the measurement of **a**–**c** and **k** current–voltage curve for the measurement of **d**–**f**. The highlighted turning points in **j** and **k** correspond to the snapshots of **b**, **e** above, respectively. Scale bars in **a**–**f** = 5 nm.

The effect of crystal defects can be further demonstrated by the in situ formation/escape of dislocation in the exfoliated MoS_2_. In two sequential 6L edge contact (edge-on view) measurements, a screw dislocation was observed to emerge from perfect layers (Fig. 5a–c, 17 s, Supplementary Movie 2), motivated by the tip movement and local shear force. The excess layer edge on the top after half unit cell slip (also burger’s vector = 1/2**z**) through the layers during screw dislocation formation could be visualized (Fig. 5b, c). The screw dislocation existed for 6 s and then disappeared and probably escape from the surface (edge for 2D) with the further tip movement (Fig. 5d–f, 3 s, Supplementary Movie 3). The contact zone was totally recovered after the elimination/escape of the screw dislocation. The Supplementary Movies 2 and 3 present this entire process. The atomic model and TEM image simulation by multislice method. Figure 5g–i was built and tested for the screw dislocation core, the result coincides with our in situ TEM observations. Although the formation of screw dislocation in vdW materials usually requires quite high energy, the case we observed are very close to the free edge which can considerably lower down the energy cost. Actually, the screw dislocations were widely found in 2D vdW structures grown by CVD method^44^.

Two *I*–*V* measurements (±2.00 V scan) were simultaneously carried out through the above process. It can be seen the conductance was abruptly enhanced when the screw dislocation was formed. In specific, the conductance was risen from 2.00 to 3.88 nS (Fig. 5j). Vice versa, when the screw dislocation annihilated, the conductance returned to the origin, decreased from 4.81 to 2.39 nS (Fig. 5k). Thus, the screw dislocation increased the contact conductance by nearly 100%, which is in strong agreement to our in situ electrical measurements on single screw dislocations^44^. Because of the screw dislocation, all the six atomic layers were connected and contributed to the transport, rather than relying on the vdW interactions and electron tunneling in perfect multilayer MoS_2_ (Supplementary Fig. 10). The conductance enhancement by screw dislocation was widely found in many other materials^50–52^, however, much higher conductance was gained by single dislocation in 2D contacts due to the change from vertical transport into in-plane transport from separated 6 layers to the inter-connected six layers.

In situ TEM-STM experiments provided rich and fundamental insights for the electrical contacts with 2D materials. Distinct to the bulk contacts, due to the large vdW surfaces in 2D materials, the current is mainly attributed to the field emission through ultra-thin potential barriers, via direct tunneling or FN tunneling. Direct tunneling is rendered when the contact area is smaller, while FN tunneling governed the transport when the contact area is larger. A critical area of 249 and 97.0 nm^2^ corresponding to DT-FN transition is found for the contacts with 2D MoS_2_ and ReS_2_, respectively. Almost “Ohmic contacts” and much higher conductivities in vdW-type contacts suggested the favorable contact modes with 2D materials in future applications.

## Methods

### In situ TEM-STM

JEOL 2100F TEM with 200 kV electron energy and Nanofactory in situ STM-TEM holder were employed in this experiment. *I*–*V* data were collected by Keithley 2400, which is controlled by a LabVIEW program (Supplementary Fig. 11). The experimental setup was shown in Fig. 1a. The piezoelectric-driven fine control provides accurate control of probe to contact with different sample (maximum range: ±14 µm; minimum step: 2 pm). For example, in the Supplementary Movie 1, the tip was moved to the left and then touched the MoS_2_ flake, which was bended into be edge-on configuration, forming the face contact. If the tip was moved a little bit rightward and then let the edge of the flake contact with the W tip, that contact would become edge contact. Other than the above bending manipulation and TEM contrast changes, electrical current response also can indicate the successful formation of contact (Supplementary Fig. 12). After the stable contact was formed, a pulse would be generated each 0.5 s with 150 µs pulse width and the data would be collected simultaneously to minimize the accumulation of joule heating. During experiments, the area of hydrocarbon contamination/residue on the 2D material surfaces can be easily excluded by the W tip and manipulator, because the contact resistances at the contaminated zones will be much higher than the clean zones.

All the in situ TEM results were collected approximately in room temperature. The joule heating of the current density is insignificant, using the reported thermal conductivities of MoS_2_^53^, the temperature rise by joule heating is estimated below 10 K, even under ±5.00 V measurements. Moreover, our pulsed instead of static electrical measurement further minimized the joule heating effect. Regarding the beam damage effect, the TEM 200 kV beam damage for the MoS_2_ is mainly knock-on damage^54,55^, followed by radiolysis and heating. However, the electron dose has been maintained under 1 cm^−2^ throughout the in situ experiments. No apparent heat damage was discovered on the samples (Supplementary Fig. 13), while the electrical transport results were highly reproducible which also demonstrate the minimized beam damage. The bended part in red dot circle was created by the additional stress tests. Therefore, the defects involved in our experiments were mainly intrinsic or induced by strains and W tip manipulations. For the face contacts with CVD grown samples, we have also tested the compressive strain dependence. A slight compressing force onto the free-standing specimens can be applied by the W tips. Schematic is shown in Supplementary Fig. 14. This pN-to-nN scale compressive force rendered significant changes in contact resistance.

### Specimen preparations

1L graphene was synthesized on copper foil (Goodfellow, England) using tube furnace (Nabertherm, Germany) with a flow of 1000 sccm Ar and CH_4_/H_2_ (ratio of 1:5) for 45 min at 1060 °C in atmospheric pressure. 1L ReS_2_ was grown on a c-face sapphire substrate by the atmospheric CVD system. Ammonium perrhenate (NH_4_ReO_4_) (Aldrich, 99.999 %) and Sulfur powder (Aldrich, 99.998 %) were used as precursors with weight ratio 1:50, separately put in two quartz boats. A two-zone splitting tube furnace was used to control accurately Sulfur and substrate zone temperature, respectively. Prior to the temperature ramping up, 300 sccm of Argon gas was purged through the quartz tube for 10 min. During the deposition process, argon gas (80 sccm) was as the carrier gas to transport sulfur vapor to substrate zone. The 1L MoS_2_ was grown by CVD in similar fashion. The few layer MoS_2_ was fabricated by mechanical exfoliation from a bulk crystal of MoS_2_. The few layer MoS_2_ flakes on scotch tape was transferred to the quantifoil TEM grid by thermal release tape method^56^. The tested specimens were annealed at 150 °C in ultra-high vacuum for at least 12 h before the experiments.

### Tungsten tip preparation

Tungsten tip was fabricated by chemical etching method^57^ (Supplementary Fig. 15). 1 M NaOH electrolyte was implemented to etch tungsten wire. A 6.0 V bias voltage was applied for the reduction reaction. The tip would be gradually thinner and finally drop into collector. The tip would be further cleaned by ethanol and deionized water. The excess liquid on the tip would be removed by dust cleaner balloon. W tips were freshly made before the experiments.

### *I*–*V* data processing

Origin software was used for the data processing. The in situ *I*–*V* measurements mainly described the tip-to-sample behavior. The current path of our experiment can be seen in Supplementary Fig. 16. The main contributions of the voltage drop are *R*_S_ and *R*_Contact_. The resistance of the MoS_2_ flake is around 1.6 × 10^3^ to 6.4 × 10^5^ Ω^58,59^, which is much smaller than the total resistance (>10^8^ Ω). On the other hand, the interlayer resistance of the MoS_2_ flake is at most 5.1 × 10^4^ Ω^60^. Besides, although the actual current path inside the MoS_2_ is unknown, the current must go through the path with the lowest resistance. Therefore, the contribution of the contact is dominant in the whole circuit and we can assume the *I*–*V* curve solely represents the contact behavior. The 6-layer (6L) MoS_2_ for edge contact and face contact were measured in a range from −5.00 to 5.00 V. The majority measurements behave Ohmic within ±2.00 V. Compared to the typical thermionic emission and thermionic field emission curve, the Ohmic contacts obey field emission (FE) (Supplementary Fig. 17). When the bias voltage is larger than 2.00 V, the temperature of the junction is slightly increased due to joule heating. The conductivity of semiconductor is increased with temperature and hence the turning point occurs at high bias (around ≥2.00 V). Since the contribution of heat is low, the *I*–*V* data were directly used for simplifying the calculations. For the FE, there are two common tunneling models, which are direct tunneling model and FN tunneling, which can be distinguished into two groups by contact area. The contact area was estimated by ImageJ. In terms of the small area contacts (<50 nm^2^), direct tunneling was dominant in our results. The linear relationship between ln(*I*/*V*^2^) and ln(1/*V*) can be demonstrated as below^31^,

1 $$\ln \left( {\frac{{\it{I}}}{{{\it{V}}^2}}} \right) \propto \ln \left( {\frac{1}{{\it{V}}}} \right) - \frac{{4{\it{\uppi }}d\sqrt {2{\it{m}}_{\rm{e}}^ \ast \varphi } }}{{\it{h}}},$$

where *I* is the current flow across the contact, *V* is the applied bias, *d* is the barrier width/vdW gap, $m_{\mathrm{e}}^ \ast$ is the electron effective mass (0.37*m*_e_ for MoS_2_^61^ and 0.5*m*_e_ for ReS_2_^62^), *φ* is the potential barrier, and *h* is the Planck constant. In terms of large area contact (>95 nm^2^), Fowler–Nordheim tunneling became dominant, which is denoted by^32^,

2 $$\ln \left( {\frac{{\it{I}}}{{{\it{V}}^2}}} \right) = \ln \left( {\frac{{{\it{a}}A}}{{\lambda d^2\varphi }}} \right) - \frac{{\nu b\varphi ^{\frac{3}{2}}d}}{{\it{V}}},$$

where *a* and *b* are the first and the second Fowler–Nordheim constant respectively, *A* is the estimated contact area, *V* is the bias voltage, *d* is the thickness (barrier width for this case), *φ* is the potential barrier height, *λ* is local pre-exponential correction factor, and *ν* is the correction factor for the triangular barrier. In this experiment, *λ* and *ν* are 0.005 and 1, respectively. The barrier height and width can be found by solving the simultaneous equation eventually. The contact resistance (*R*) was denoted by,

3 $${\it{R}} = \left[ {\frac{{{\rm{d}{\it{V}}}}}{{{\rm{d}{\it{I}}}}}} \right]_{{\it{V}} = 0}.$$

By the fitted *d* and *R*, the contact resistivity can be also estimated by,

4 $$\rho = \frac{{{\it{R}} \times {\it{A}}}}{d},$$

where *ρ* is the contact resistivity, *A* is the estimated contact area, and *d* is the barrier width.

The temperature effect of in situ TEM experiment is mainly caused by joule heating and the irradiation heating. The maximum change of temperature by joule heating mainly depends on the electrical and thermal conductivity, and the equation is denoted by^63^,

5 $$\Delta {\it{T}} = \frac{{\sigma V^2}}{{8\kappa }},$$

where *σ* is the electrical conductivity, *V* is the applied bias, and *κ* is the thermal conductivity. On the other hand, the maximum temperature change by irradiation heating is^64^,

6 $${\mathrm{\Delta }}{\it{T}} = \frac{{{\it{I}}_{\rm{b}}}}{{4\pi \kappa q}} \times \frac{{{\mathrm{\Delta }}{\it{E}}}}{{\it{t}}}\left[ {1 + \ln \left( {\frac{{{\it{r}}_{\rm{s}}}}{{{\it{r}}_{\rm{e}}}}} \right)} \right],$$

where *I*_b_ is the beam current, *q* is the electron charge, Δ*E* is the total energy lost per electron, *t* is the thickness of sample, *r*_s_ is the radius of sample, and *r*_e_ is the radius of electron.

### Contact area measurements

For the monolayer samples, the (face) contact area was measured by using the radius of curvature (*r*) of the W tip (round shape tips with large *r* and without faceting are chosen for face contact measurements) and the indentation depth (*d*) (2 nm, as controlled by the in situ TEM manipulator) of the sample along normal direction. The contact structure can be seen in Supplementary Fig. 18. Under small deformation approximation ($d < < r$), the contact between the tip and 2D materials can be calculated by using an inclined (inclination angle *θ*) indentation model^65^, the contact area is denoted by,

7 $$a = \sqrt {rd} = \sqrt {rz{\rm{cos}}\theta },$$

8 $${\mathrm{Contact}}\;{\mathrm{area}} = \pi r^2\left( {{\sin} ^{ - 1}\frac{{\sqrt {z{\rm{cos}}\theta } }}{{\sqrt r }}} \right),$$

where *r* is the radius of curvature of the tungsten tip, *z* is the movement of the tip along *z*-direction, and *θ* is the inclination of the sample. On the other hand, for the few layer 2D samples, the edges for contacts are in normal direction, and the contact areas can be directly determined by the TEM images, simply measured by the product of overlapping layers and interlayer spacing (0.615 nm) by using ImageJ (Supplementary Figs. 19, 20 and Supplementary Note 3).

### Density functional theory calculation

Spin-polarized DFT calculations are carried out using the Vienna ab initio simulation package (VASP) program package^58^ within the projector augmented wave (PAW) to explore geometries and electronic properties of MoS_2_. The exchange-correlation functions are described with the generalized gradient approximation (GGA) in the form of the Perdew, Burke, and Ernzernhof (PBE) functional^59^. The DFT-D3 scheme of Grimme for the vdW correction^60^ is applied on multiple layers of MoS_2_. The whole systems contain 71–432 atoms. The kinetic energy cutoff for the plane-wave basis set is chosen as 450 eV, and the distance of vacuum layer is set to be more than 20 Å, which is sufficient large to avoid interlayer interactions. The electronic SCF tolerance is set to 10^−5^ eV, and dipole corrections in the *Z* direction are applied. Fully relaxed geometries and lattice constant are obtained by optimizing all atomic positions until the Hellmann–Feynman forces are <0.02 eV Å^−1^. The *k*-points samplings with a gamma-centered Monkhorst–Pack scheme^61^ are 5 × 5 × 1 for structural optimizations and the density of states calculations.

## Supplementary information

Supplementary Information

Description of Additional Supplementary Files

Supplementary Movie 1

Supplementary Movie 2

Supplementary Movie 3

## Data Availability

The data that support the findings of this study are available from the corresponding author on reasonable request.
