# Supplementary material for: Site-specific electrical contacts with the two-dimensional materials
Source: Nat Commun. 2020 Aug 7;11:3982. doi: 10.1038/s41467-020-17784-3 (PMC7414847; doi:10.1038/s41467-020-17784-3)
Supplement: Supplementary file 1 — Supplementary Information [file 41467_2020_17784_MOESM1_ESM.pdf]

Supplementary Information for

Site-Specific Electrical Contacts with the Two-Dimensional Materials

Lok-Wing Wong[1,2], Lingli Huang[3,4], Fangyuan Zheng[1,2], Quoc Huy Thi[3,4], Jiong Zhao[1,2*], Qingming Deng[5*], Thuc Hue Ly[3,4*]

[1] Department of Applied Physics, The Hong Kong Polytechnic University, Kowloon, Hong Kong, China

[2] Polytechnic University of Hong Kong Shenzhen Research Institute, Shenzhen, China.

[3] Department of Chemistry and Center of Super-Diamond & Advanced Films (COSDAF), City University of Hong Kong, Kowloon, Hong Kong, China

[4] City University of Hong Kong Shenzhen Research Institute, Shenzhen, China.

[5] Physics department and Jiangsu Key Laboratory for Chemistry of Low-Dimensional Materials, Huaiyin Normal University, Huaian 223300, China.

*email: jiongzhao@polyu.edu.hk (J.Z.), qingmingdeng@gmail.com (Q.D.), thuchly@cityu.edu.hk (T.H.L.)

This material contains 1 Supplementary Tables, 3 Supplementary Notes and 20 Supplementary Figures.

Supplementary Table 1: Summary of measurement and fitting results for 42 different contact configurations.

Test No.	No. of layer	Type of Contact	A (nm^2)	R (MΩ)	ϕ (eV)	d (nm)	R^2 (%)	ρ (MΩ µm)
1	1	Edge	3.40	131	2.46	1.67	91.9	0.267
2	1	Edge	3.49	161	2.71	1.77	90.4	0.318
3	1	Edge	3.64	317	2.52	1.70	93.2	0.680
4	1	Edge	5.74	660	1.97	1.48	94.7	2.56
5	1	Edge	4.39	526	1.87	1.44	95.8	1.61
6	1	Edge	4.37	512	1.84	1.42	97.4	1.57
7	1	Edge	4.29	495	1.80	1.41	98.9	1.51
8	1	Edge	3.95	452	2.04	1.51	98.7	1.19
9	1	Edge	3.84	478	2.17	1.56	97.6	1.18
10	2	Edge	8.98	447	2.09	1.53	97.0	2.63
11	2	Edge	7.49	784	2.03	1.51	96.7	3.90
12	2	Edge	6.86	463	1.83	1.42	97.1	2.24
13	2	Edge	9.95	647	1.91	1.46	98.8	4.42
14	2	Edge	7.14	343	2.13	1.54	97.1	1.59
15	2	Edge	5.34	329	2.20	1.57	98.2	1.12
16	3	Edge	6.66	382	2.16	1.56	97.6	1.64
17	6	Edge	21.2	333	2.05	1.51	96.9	4.69
18	6	Edge	39.8	295	2.18	1.56	98.0	7.50
19	6	Edge	37.7	274	2.13	1.54	98.0	6.71
20	6	Edge	37.8	275	2.15	1.55	97.7	6.72
21	1	Face	17.8	519	2.61	1.73	95.6	5.33
22	1	Face	18.8	613	3.39	2.02	90.9	5.71
23	2	Face	15.6	174	2.04	1.50	98.7	1.81
24	2	Face	20.2	176	2.06	1.51	98.9	2.34
25	2	Face	18.5	172	2.05	1.50	98.8	2.12
26	2	Face	27.8	172	2.22	1.57	97.3	3.04
27	6	Face	35.7	558	2.34	1.63	96.6	12.2
28	6	Face	21.3	452	1.75	1.38	98.7	6.94
29	6	Face	18.4	471	1.85	1.43	98.1	6.07
30	6	Face	18.1	409	1.80	1.40	98.8	5.26
31	6	Face	42.5	538	2.28	1.60	94.4	14.3
32	6	Face	42.2	387	2.02	1.50	96.4	10.9
33	6	Face	44.4	379	2.02	1.50	97.1	11.2
34	6	Face	27.6	345	2.07	1.52	98.3	6.28
35	6	Face	29.1	342	2.13	1.54	97.8	6.45
36	6	Face	24.2	331	2.13	1.54	98.2	5.19
37	6	Face	21.4	207	2.06	1.51	98.5	2.94
38	6	Face	23.2	209	2.15	1.55	97.8	3.14
39	6	Face	44.7	199	2.08	1.52	98.7	5.87
40	6	Face	41.8	129	2.13	1.54	97.5	3.50
41	6	Face	41.8	125	2.09	1.52	97.7	3.44
42	6	Face	48.8	121	2.12	1.53	97.6	3.86

Supplementary Figures and Captions

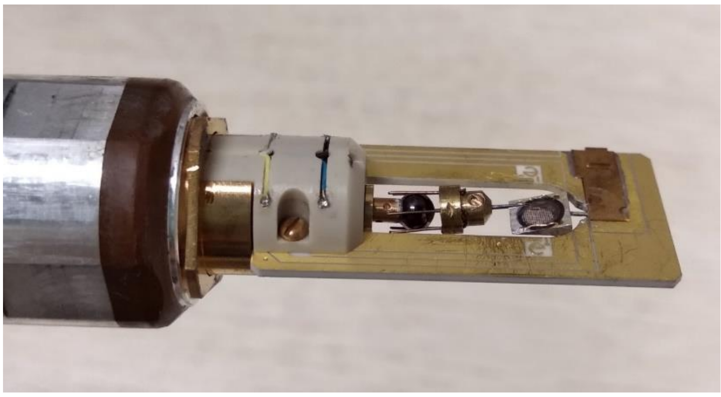

Supplementary Figure 1. Images of the TEM-STM setup. An additional homemade grid supporting stage was designed and fabricated. The inclination of the grid supporting stage is 15º-30º.

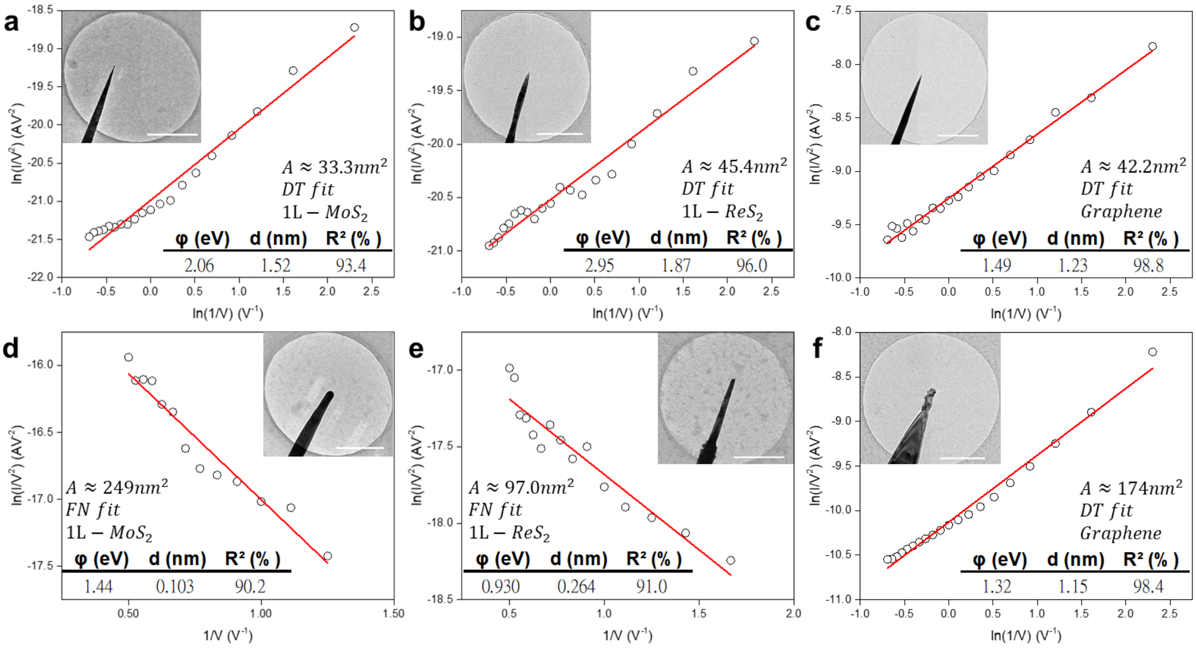

Supplementary Figure 2. DT and FN characteristic fits of (**a-c**) small contact areas and (**d-f**) large contact areas with respect to MoS_2, ReS_2, and graphene, respectively. Scale bar: 0.5 μm.

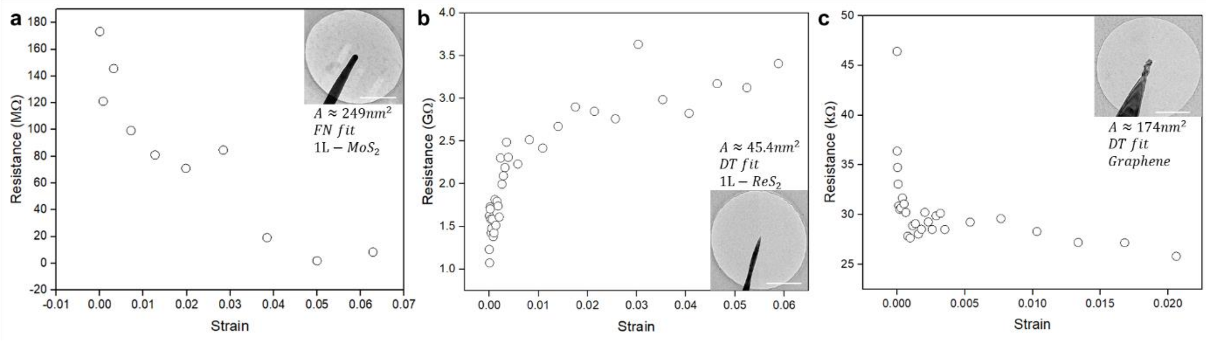

Supplementary Figure 3. Resistance versus Strain curves of (**a**) MoS_2, (**b**) ReS_2 and (**c**) graphene.

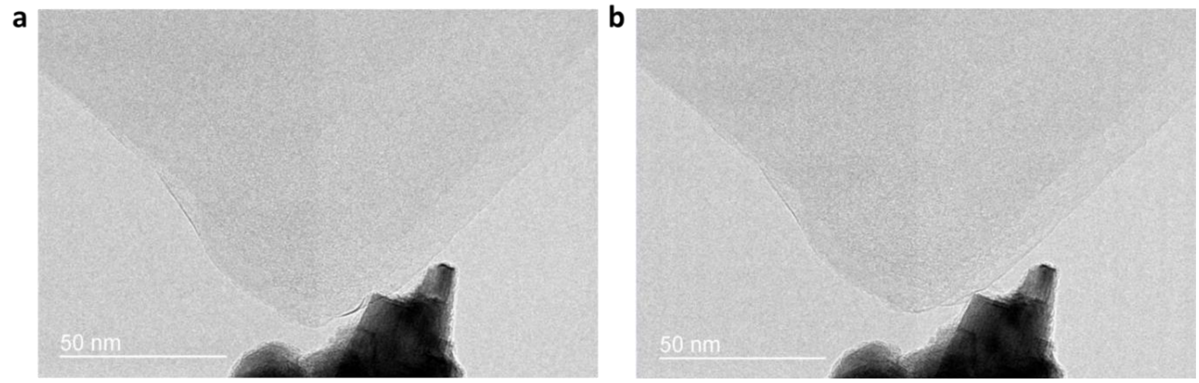

Supplementary Figure 4. TEM images captured from video **Supplementary Movie 1**. (**a**) Face contact forms at 0:05. (**b**) Edge contact forms at 0:14.

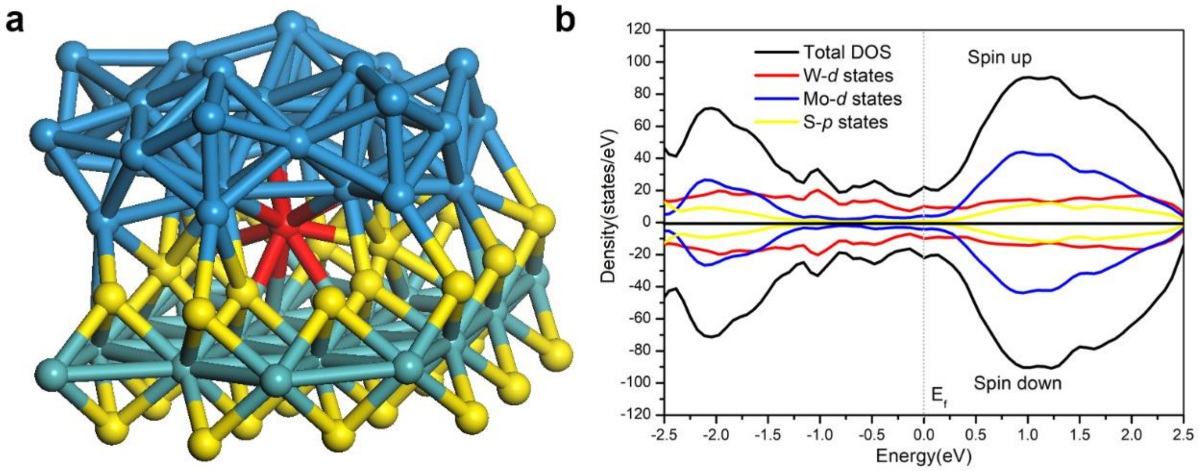

Supplementary Figure 5. (**a**) the DFT optimized model for W (blue) and MoS_2 (green and yellow) interfaces with a S vacancy. The closest W atom (red) resides right on the S vacancy and creates the strongest chemical interaction. (**b**) The DFT calculated density of state (DOS) of W/MoS_2 interfaces with S vacancy.

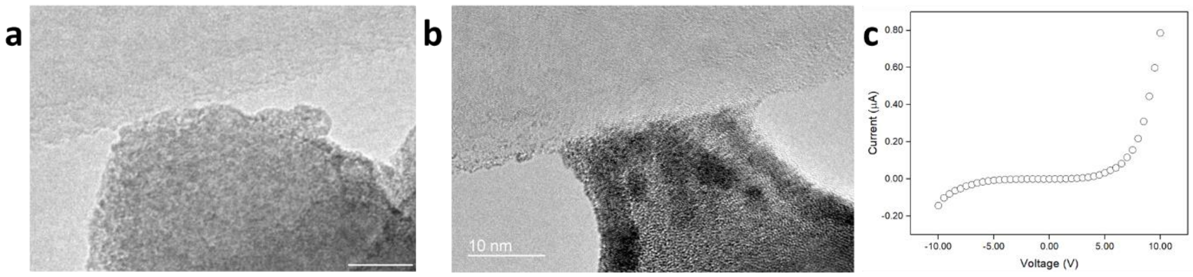

Supplementary Figure 6. *In situ* TEM images for (**a**) before and after (**b**) 10 V bias voltage measurement between a few layer MoS_2 flake and W tip. **c** I-V curve after welding.

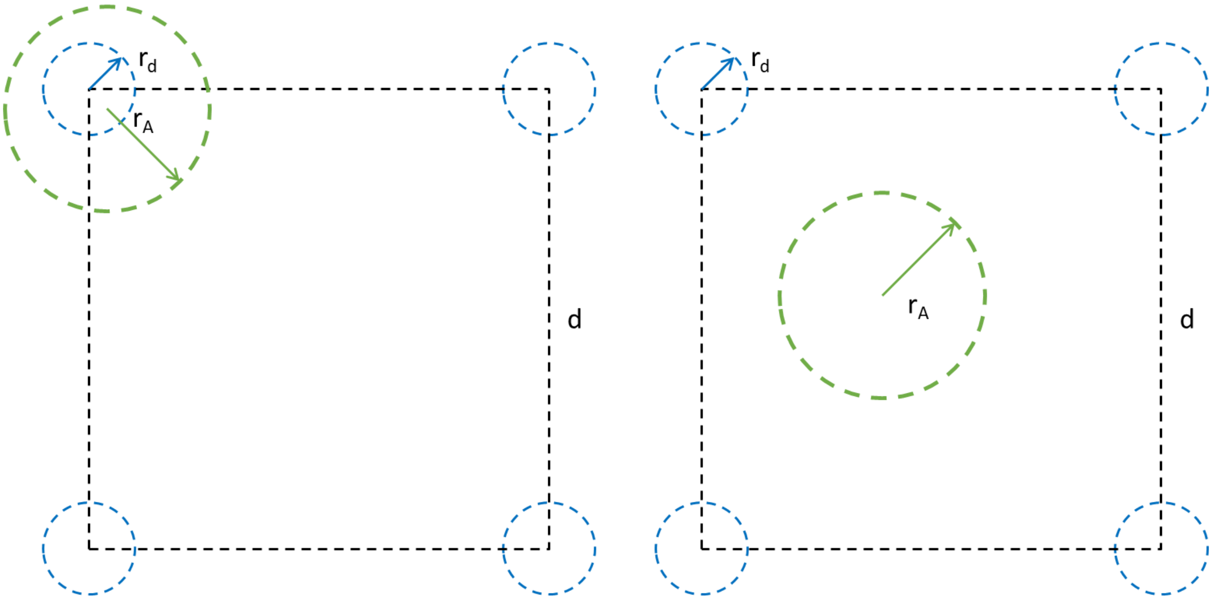

Supplementary Figure 7. Schematic of the contact with defects and non-defective areas.

Supplementary Note 1

The Fermi level pinning at the defective states deep in the bandgaps can easily trigger FN barriers at the contacts (for 2D TMDs which are usually n doped). In the revised manuscript, we have added explanations for the area dependent contact behavior. We can assume the defects are homogeneously distributed in the 2D sample by using the defect density 10^{12} cm^{-2}. The separation of defects d = 10 nm. The Debye screening length (r_d) in 2D MoS_2 is around 1.5 to 2.0 nm^2. Therefore, the defect sites will possibly not be included in the contact area if the contact area is sufficiently small, while larger contact area will have tendency to include the defect sites. Assume all of our contacts cover circle area with a radius (r_A) on a plane as Supplementary Figure 7, the averaged conductivity is denoted by,

$$\sigma = \begin{cases} \sigma_0 - \dfrac{\pi r_A^2}{d^2}(\sigma_0 - \sigma_d) & when\ r_A < \dfrac{1}{2}d \\ \sigma_0 - f(r_A)(\sigma_0 - \sigma_d) & when\ \dfrac{1}{2}d < r_A < \dfrac{\sqrt{2}}{2}d \\ \sigma_d & when\ r_A > \dfrac{\sqrt{2}}{2}d \end{cases}$$

where σ_d is the conductivity affected by defects and σ_0 is the conductivity of perfect sample, $f(r_A) = \frac{\pi r_A^2 + 2d({r_A}^2 - d^2/4)^{1/2} - 8{r_A}^2(\arccos(d/2r_A))}{d^2}$, the contact area is thereby πr_A^2, and the area dependence can be explained in this way.

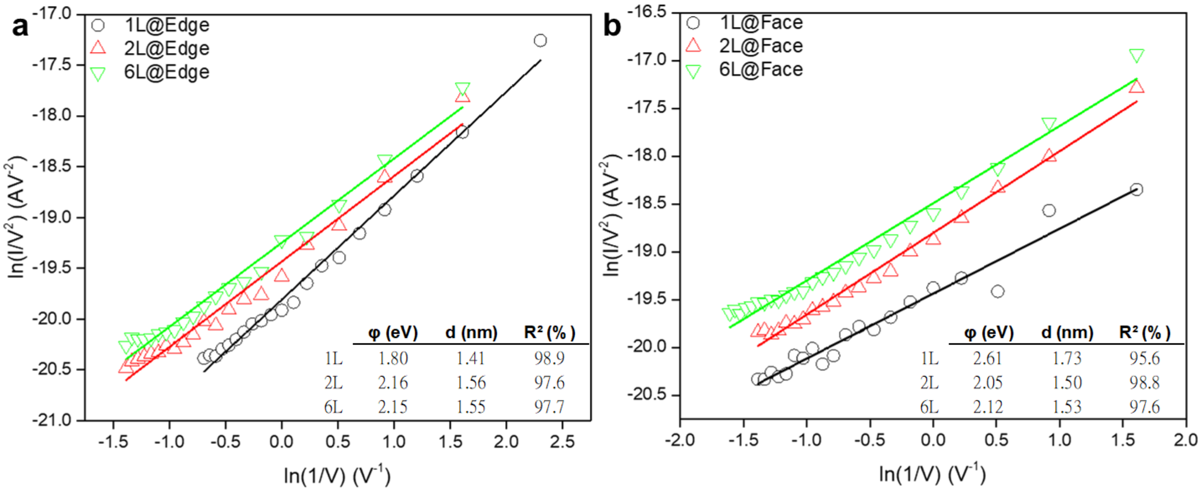

Supplementary Figure 8. DT characteristic fit of exfoliated MoS_2 for the small contact (< 50 nm^2) with respect to (**a**) edge contact and (**b**) face contact.

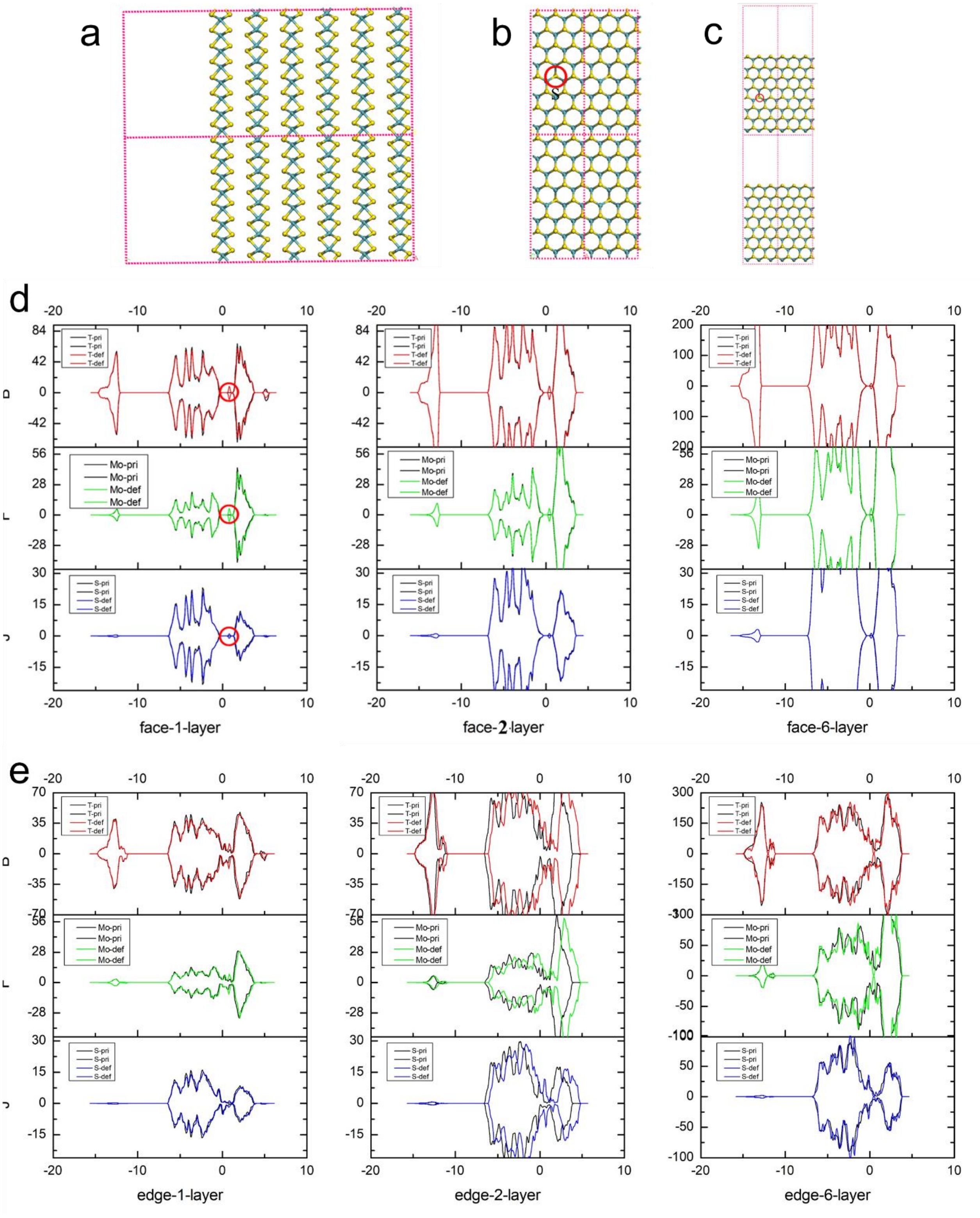

Supplementary Figure 9. DFT calculations for exfoliated MoS_2 (1L, 2L and 6L) edge contacts and face contacts. (a) The structure of pristine MoS_2. The structure of **(b)** face contacts and **(c)** edge contacts. Red circles indicate a single S-vacancy. S atoms in p orbital and Mo atoms in d orbital. DFT calculations for **(d)** face contacts and **(e)** edge contacts in terms of 1L, 2L and 6L. T is the total density of state.

Supplementary Note 2

According to Supplementary Figure 9d, compared to the pristine case, the defect states for 1L, 2L and 6L in face contacts are nearly the same. However, there is an additional peak near the fermi level, which consists of S and Mo. Since, the contribution of d orbital is larger than that of p orbital, the bandgap becomes smaller and hence the activation energy increases. In terms of edge contacts, the situations are similar (Supplementary Figure 9e). However, in the 2L and 6L case, the symmetricity of defect leads the shift of the density of state was observed. Moreover, the shift in 2L is much larger than that in 6L because of the energy level.

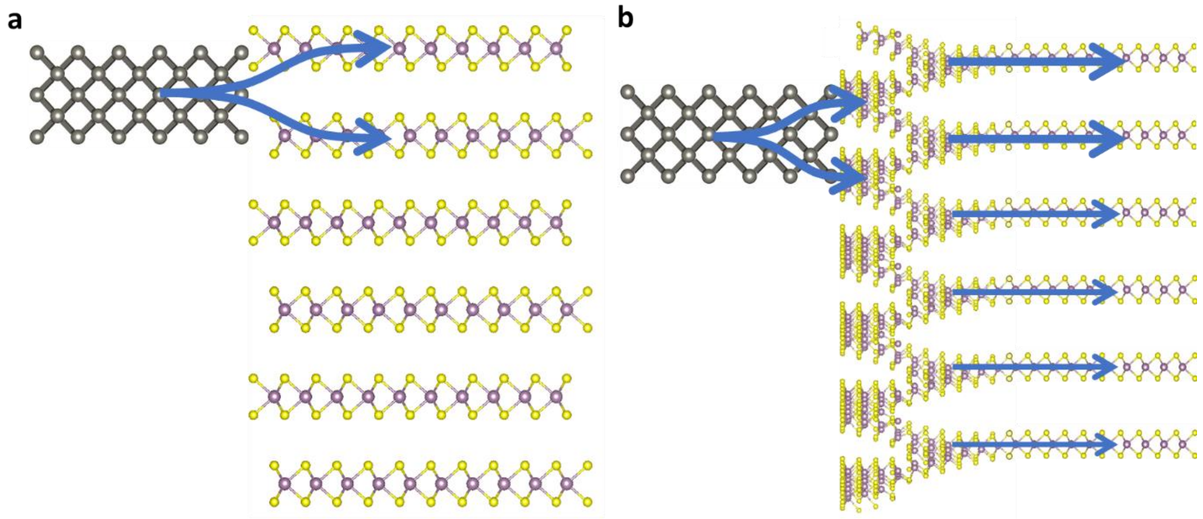

Supplementary Figure 10. Illustrations of current flow through the W-2L-MoS_2 edge contact (**a**) before and (**b**) after screw dislocation moved to the contact. Let each layer contains the same resistance R. For the original 2L edge contact, the total resistance is R/2. After screw dislocation generated, the resistance would be decreased to R/6. Therefore, the conductivity should be 3 times than the original, but the conductivity is just about twice in our data. That is because the lattice distortion and defects generated by the force of tip or/and irradiation, which increases the resistance of the circuit.

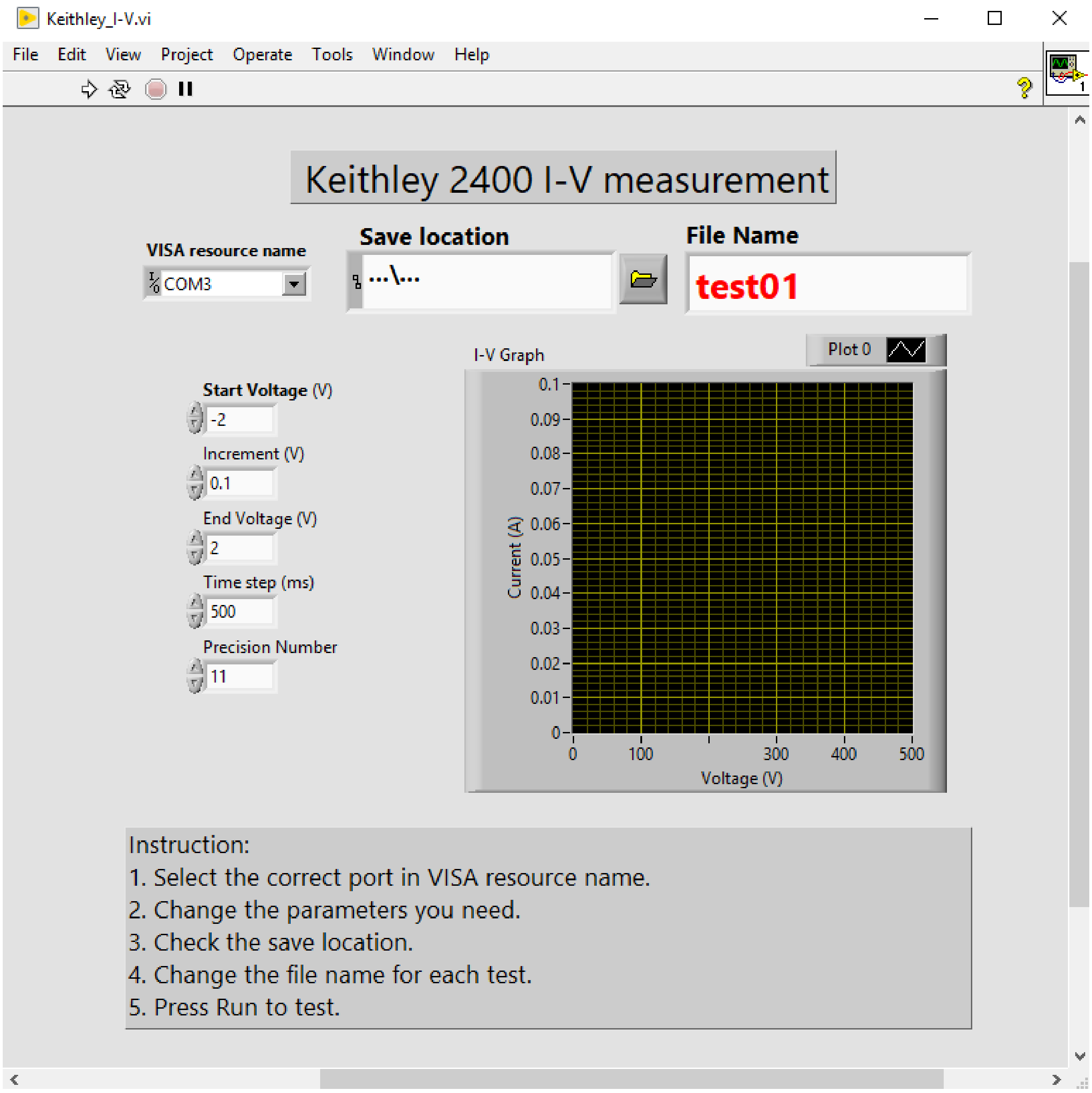

Supplementary Figure 11. Homemade LabVIEW program for I-V measurements.

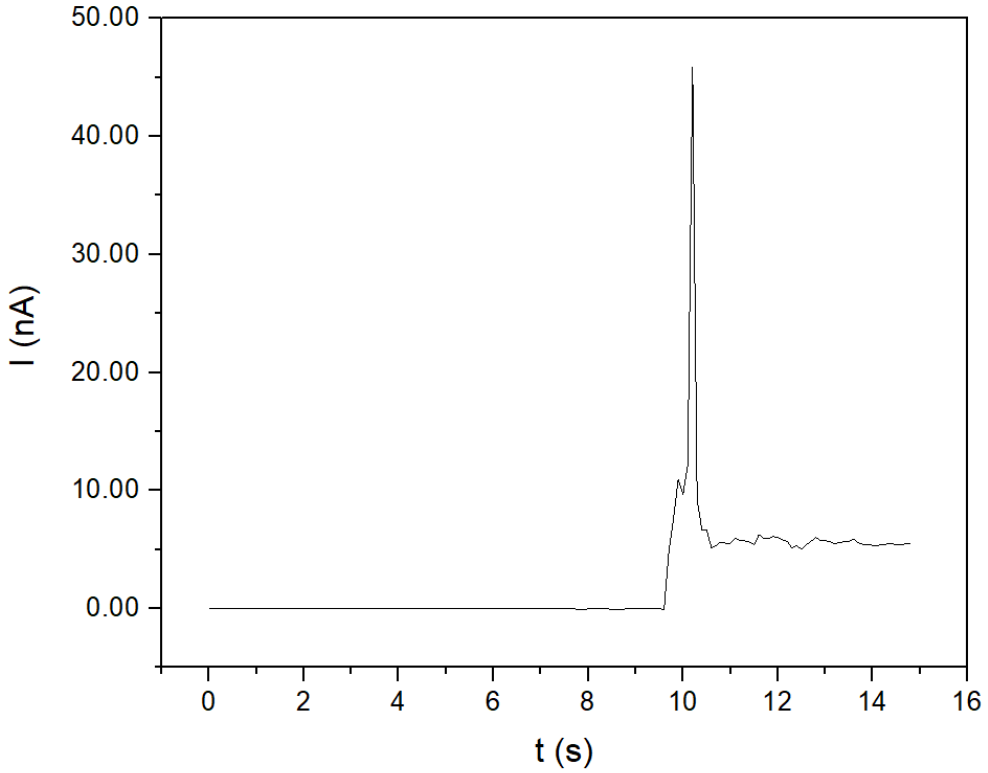

Supplementary Figure 12. Current-time plot at 1.5 V bias during controlling W tip. The contact occurred at around 10 s when the tip slowly approached to the sample.

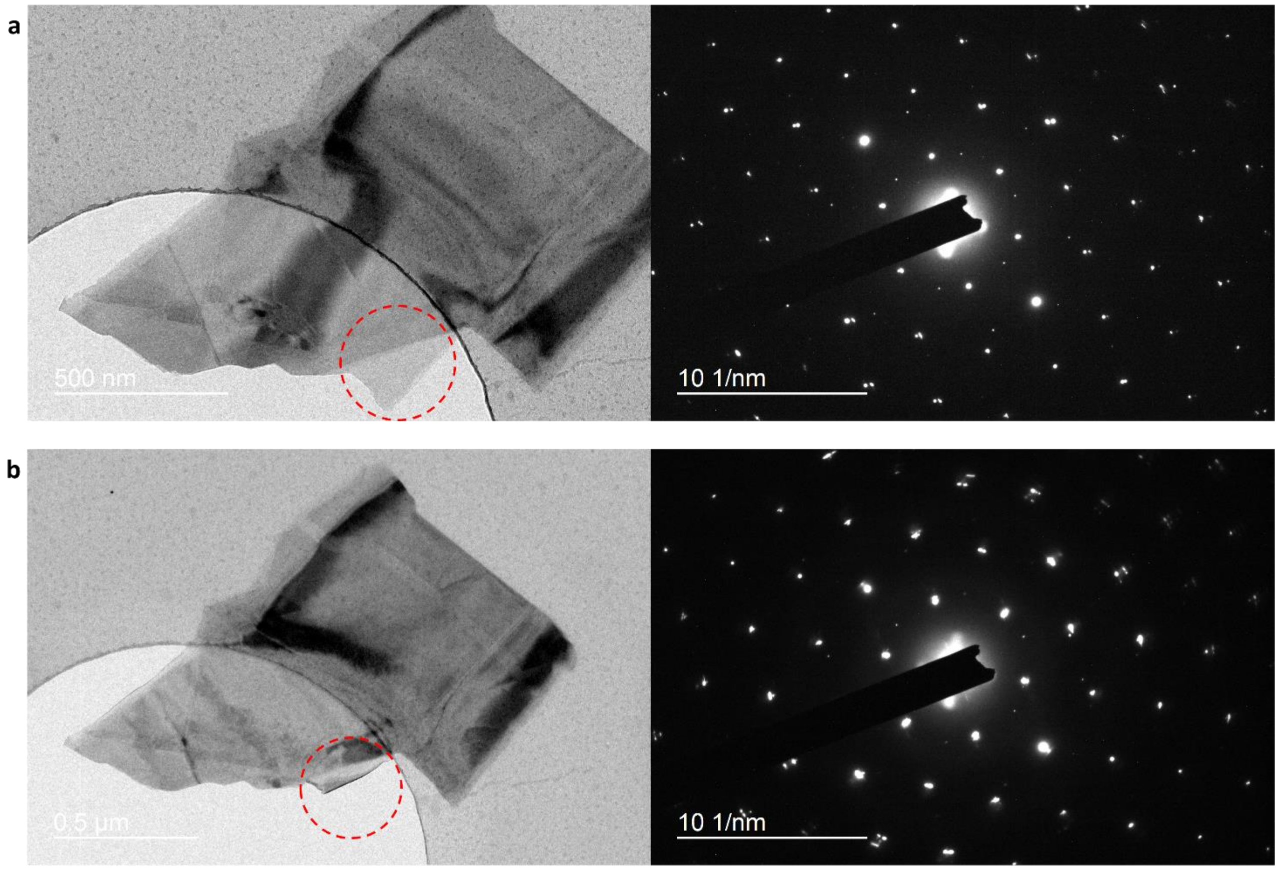

Supplementary Figure 13. TEM images of exfoliated MoS_2 with corresponding selected area electron diffraction (SAED) (**a**) before and (**b**) after measurements. The red dash circle indicates the selected area and the measurement zone.

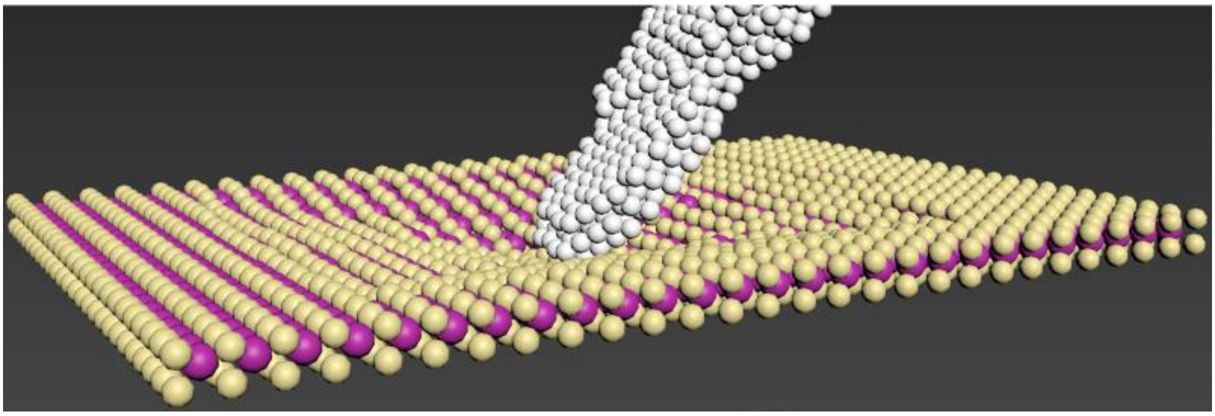

Supplementary Figure 14. Illustration of compressing force tests on face contact with 2D materials by in situ TEM-STEM method.

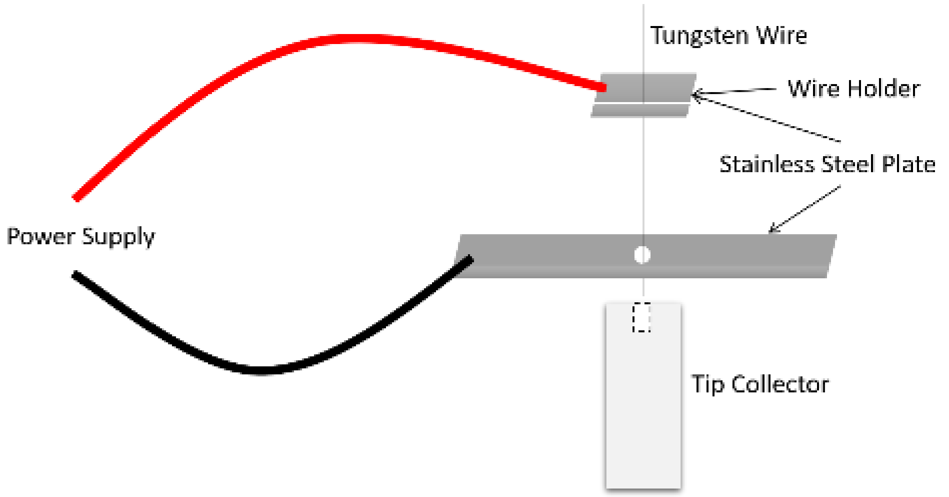

Supplementary Figure 15. Setup of the chemical etching for W tip fabrication.

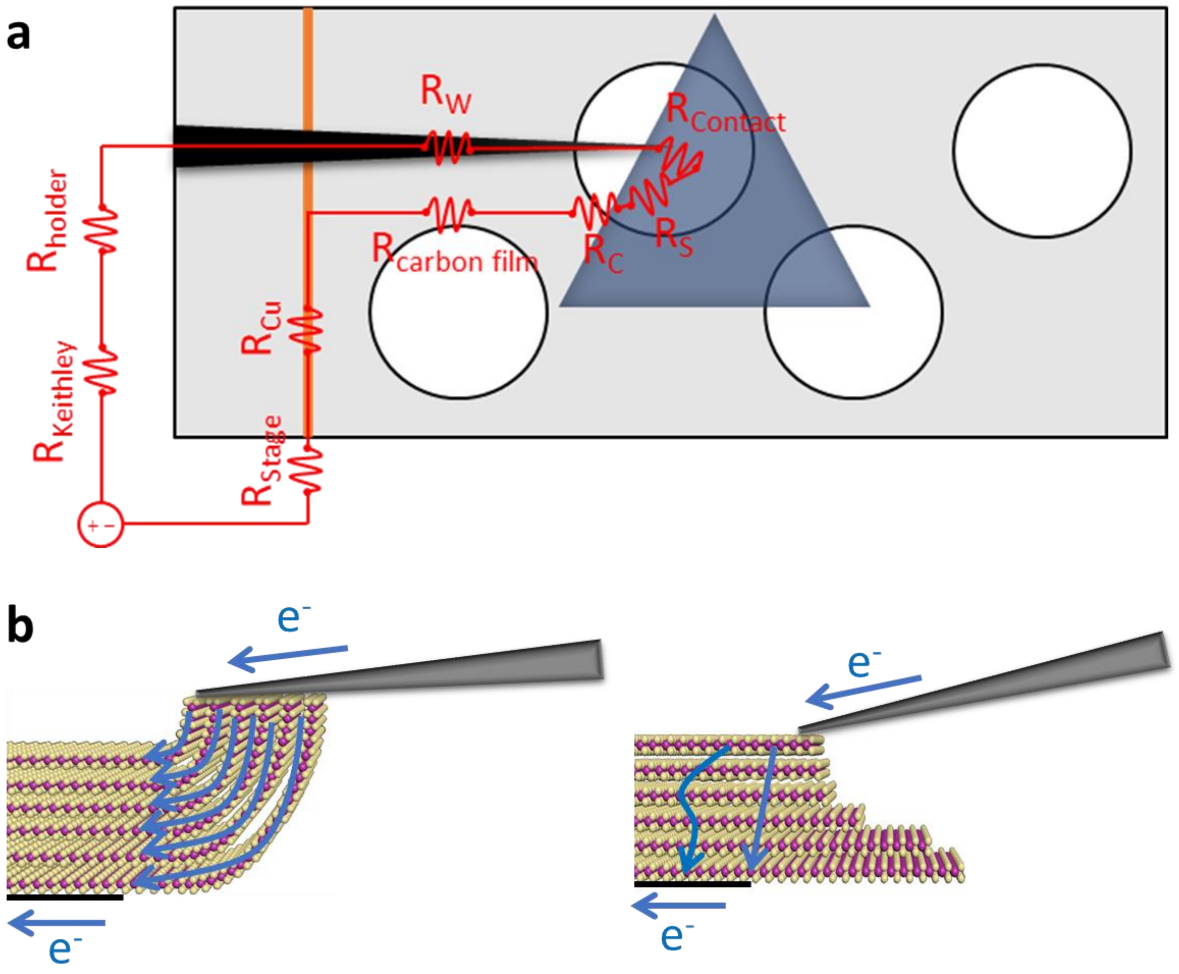

Supplementary Figure 16. (**a**) The circuit of the experiment. (**b**) Side view of the electron flow from the tip to the carbon film.

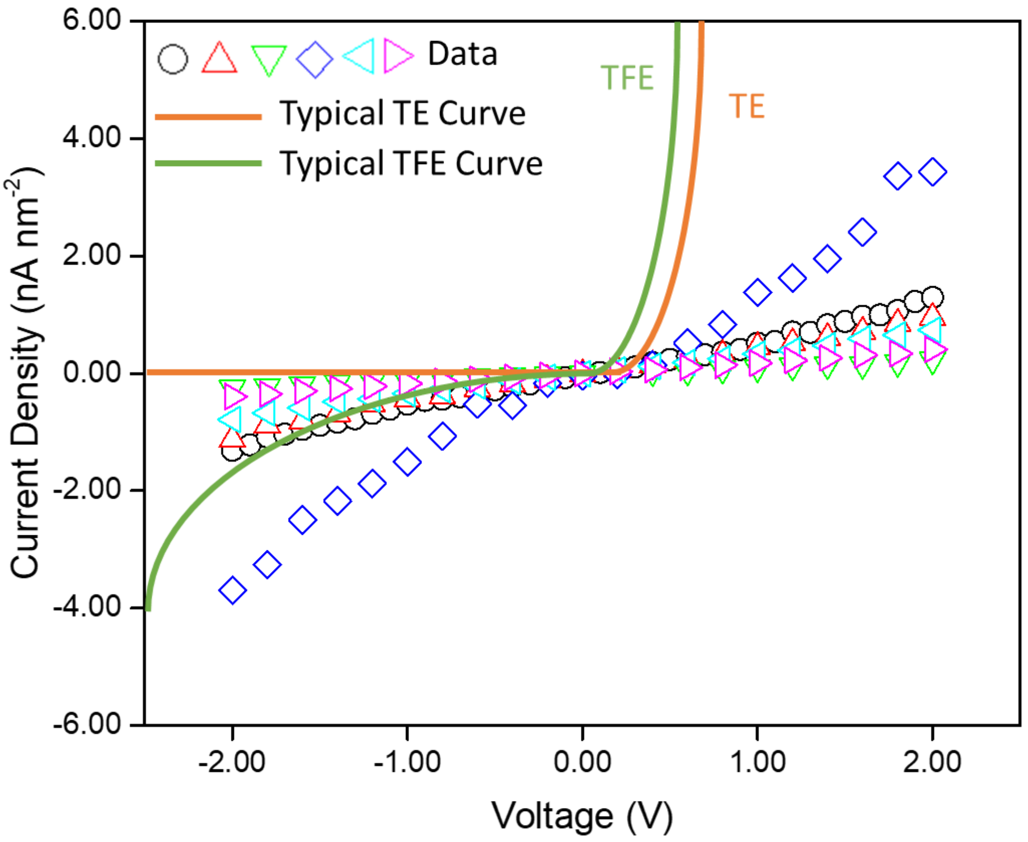

Supplementary Figure 17. Typical IV behavior of thermionic emission (TE) and thermionic field emission (TFE) compared with our data.

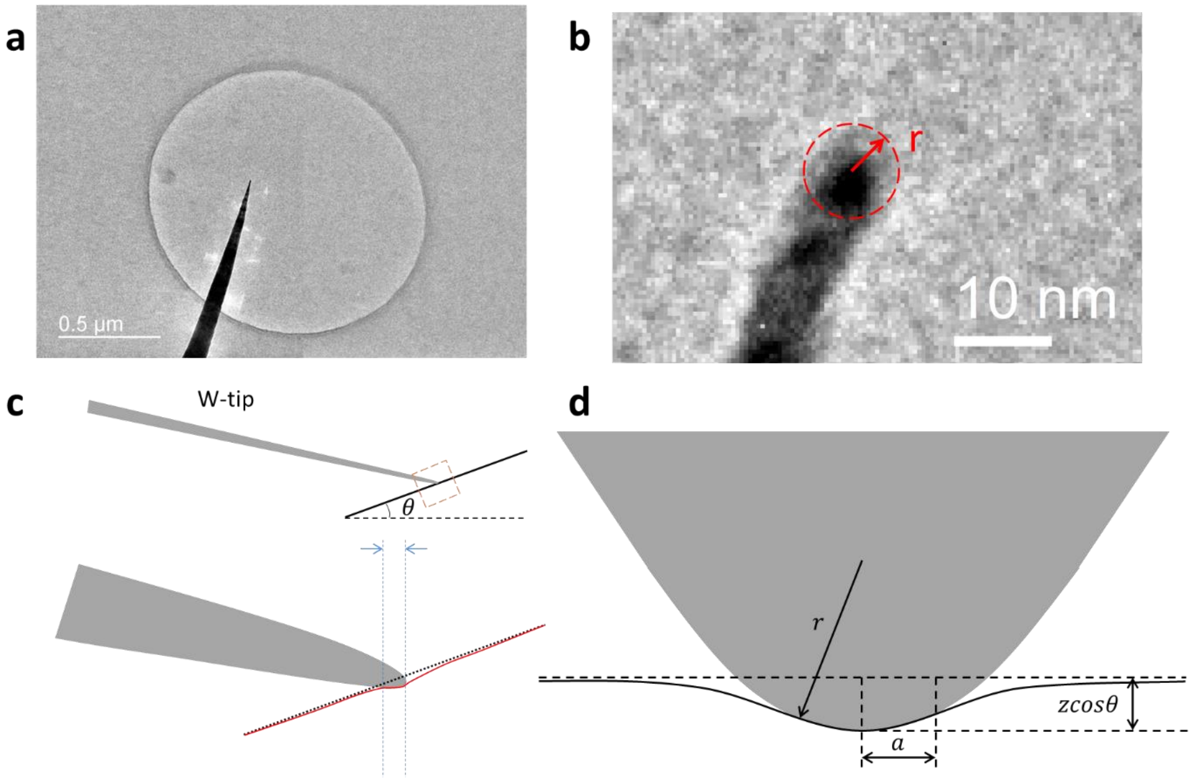

Supplementary Figure 18. (**a**) Representative TEM image of the monolayer contact. (**b**) Enlarged image of the monolayer contact. Red dashed circle indicates the radius of curvature. (**c**) Side view of the scheme showing the contact area. (**d**) Schematic diagram of the contact area.

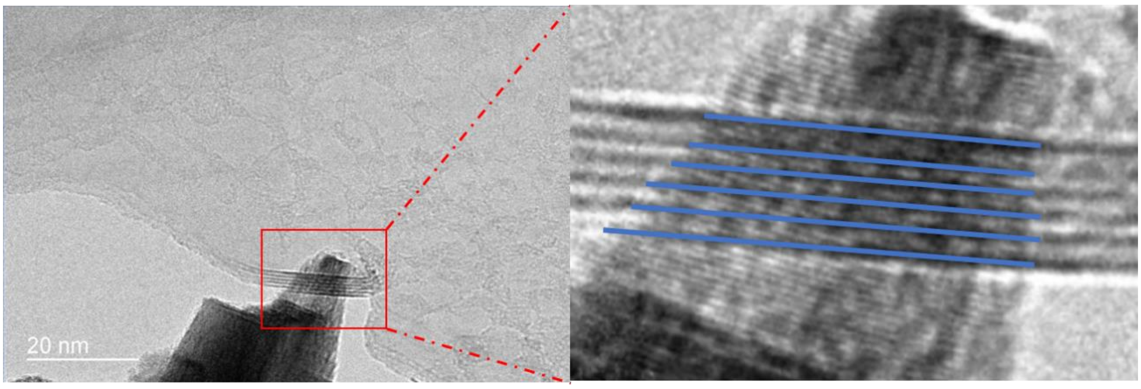

Supplementary Figure 19. The contact area of the edge contact. Blue line is the measured lengths of the contact.

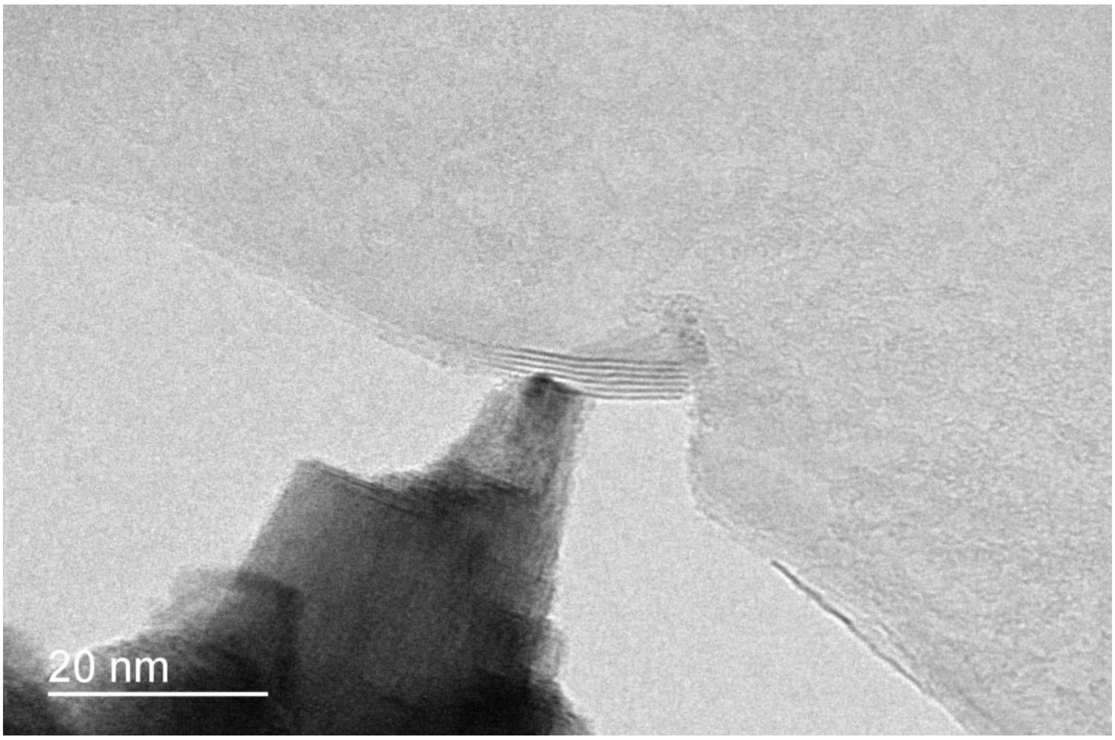

Supplementary Figure 20. The W tip contact with one layer among the 6 layer MoS_2 flake on edge.

Supplementary Note 3

To avoid the problems of improper area normalization, before the few-layer contacts are made, the edges with atomic roughness and without steps on edges are selected for electrical measurements. Hence, for the edge type contacts, the contact edges of few layer MoS_2 can be atomically flat when making contacts with W tip. Moreover, say, In a 6 layer edge contact, if the contact is just made with one single layer among all the 6 layers in the MoS_2 flake, then we move the W tip, only the single layer MoS_2 which contact with the W tip will possibly be moved or bended, and the other five layer of MoS_2 will not follow, which is in contrary to our TEM observations. In all of our experiments, we have double checked that the assigned layer numbers are firmly contacted with the W tip (see Supplementary Fig. 20), and the electrical transport signals are stable and reproducible. Different positions of the flake edges have been measured, and our conclusions are based on statistical study.

Supplementary References

1 Verhagen, T., Guerra, V. L. P., Haider, G., Kalbac, M. & Vejpravova, J. Towards the evaluation of defects in MoS2 using cryogenic photoluminescence spectroscopy. *Nanoscale* **12**, 3019-3028, doi:10.1039/c9nr07246b (2020).

2 Ghatak, S., Pal, A. N. & Ghosh, A. Nature of electronic states in atomically thin

MoS(2) field-effect transistors. *ACS Nano* **5**, 7707-7712, doi:10.1021/nn202852j (2011).
