# Supplementary material for: Site-specific electrical contacts with the two-dimensional materials
Source: Nat Commun. 2020 Aug 7;11:3982. doi: 10.1038/s41467-020-17784-3 (PMC7414847; doi:10.1038/s41467-020-17784-3)
Supplement: Supplementary file 2 — Description of Additional Supplementary Files [file 41467_2020_17784_MOESM2_ESM.pdf]

Descriptions of Additional Supplementary Files

Supplementary Movie 1

Description: Example of in situ TEM measurement on the edge contact

Supplementary Movie 2

Description: In situ formation of one screw dislocation at the contact

Supplementary Movie 3

Description: Elimination of the dislocation at the contact
